# Effectiveness and safety of PD-1/PD-L1 inhibitors monotherapy in patients with endometrial cancer

**DOI:** 10.1007/s12672-024-01033-w

**Published:** 2024-05-15

**Authors:** Xiaoyan Wan, Jiezheng Huang, Liu Huang, Yibin Wang, Yiyuan Fu, Xiaolong Jin, Zheng Huang, Jian Xiong

**Affiliations:** Department of Obstetrics and Gynecology, Guangzhou Women and Children’s Medical Center, Guangzhou Medical University, 9 Jinsui Road, Tianhe District, Guangzhou, 510623 Guangdong China

**Keywords:** Ovarian cancer, THRA, Oncogene, Bioinformatics, m6A, Endometrial cancer, Programmed cell death protein 1, Programmed cell death ligand 1, Monotherapy, Immunocheckpoint inhibitor

## Abstract

**Background:**

Studies evaluating the effectiveness of immune checkpoint inhibitors (ICI) for endometrial cancer (EC) are limited. This study aimed to assess the efficacy of PD-1/PD-L1 inhibitors as monotherapy for EC by conducting a meta-analysis. The predictive significance of MMR status, a biomarker for ICI response, also required further investigation.

**Methods:**

A systematic literature search was conducted in English databases until September 2023. The analysis included objective response rate (ORR), disease control rate (DCR), adverse events (AEs), and odds ratios (OR), along with their corresponding 95% confidence intervals (CI).

**Results:**

There were twelve trials totaling 685 individuals. PD-1/PD-L1 inhibitor monotherapy resulted in an ORR for 34% (95% CI = 24–44%) of the pooled EC patients. Subgroup analysis revealed a significantly higher ORR in dMMR EC (45%) compared to pMMR EC (8%), with an OR of 6.36 (95% CI = 3.64–11.13). The overall DCR was 42%, with dMMR EC at 51% and pMMR EC at 30% (OR = 2.61, 95% CI = 1.69–4.05). Grade three or higher adverse events (AEs) occurred in 15% of cases (95% CI = 9–24%) of the pooled incidence of AEs, which was 68% (95% CI = 65–72%).

**Conclusions:**

This meta-analysis provides significant evidence for the effectiveness of PD-1/PD-L1 inhibitors as monotherapy for EC. Notably, dMMR EC patients demonstrated superior treatment efficacy with PD-1/PD-L1 inhibitor immunotherapy. Further research is required to explore subclassifications of EC based on dMMR molecular subtypes, enabling improved treatment strategies and outcomes for EC patients.

## Introduction

Endometrial cancers (EC) represent a group of malignant epithelial tumours that develop in the endometrium of the uterus. They are more commonly diagnosed in perimenopausal and postmenopausal women, and in developed countries, they now account for nearly half of the new gynaecological malignancies [1, 2]. A 5 year survival rate of approximately 17% is predicted for women with advanced EC, giving them a poor prognosis [3]. The response rate to successive treatment after progression to first-line platinum-taxane chemotherapy is only 20% or less [4]. Consequently, researchers have focused on investigating molecular pathogenesis and immunomodulation to devise novel targeted therapies.

One emerging option for advanced EC is immune checkpoint inhibitor (ICI) therapy, which has shown remarkable efficacy in treating various other tumours. Notably, anti-PD-1, also known as anti-programmed cell death protein 1, or anti-PD-L1 (also known as anti-programmed cell death ligand (1), is frequently expressed at high levels in EC; rates of this expression vary from 40 to 80% in endometrioid adenocarcinomas, 10% to 68% in plasma cancers, and 23% to 69% in clear cell carcinomas [5, 6]. The current evidence for PD-1/PD-L1 use in advanced EC stems from the KEYNOTE series of clinical trials involving pembrolizumab. When pembrolizumab was used in advanced or relapsed patients who had progressed after at least one dose of standard chemotherapy, Individuals with high levels of microsatellite instability (MSI-H) and deficiencies in mismatch repair (dMMR) showed an objective response rate (ORR) that varied from 53.0% to 57.1% [7, 8], 46.7% in high tumour mutational burden (TMB-H) patients [9], and 13% in patients with positive PD-L1 expression [10]. Subsequently, several studies have been conducted to explore additional PD-1/PD-L1 inhibitors [11, 12], including nivolumab and dostarlimab-gxly, both of which have been granted accelerated approval from the US FDA for treating adult patients with advanced/recurrent dMMR EC that has progressed or recurred after platinum-containing therapy.

Beyond single-agent ICI treatment, combination therapies involving ICIs and other treatments were tested in second-line and later settings, often including microsatellite stable (MSS)/pMMR and MSI-H/dMMR tumours. For instance, Makker compared chemotherapy with pembrolizumab + lenvatinib after at least one platinum-based chemotherapy regimen progressed and found that compared to chemotherapy, combination therapy dramatically improved both progression-free and overall survival [13]. According to subgroup analysis, dMMR EC received more benefits from pembrolizumab plus lenvatinib than pMMR EC. However, the designs and available studies for these trials could not comprehensively examine subgroup differences. Additionally, not all women with advanced EC can tolerate the combination of an ICI and a polygenic inhibitor, which is considerably more toxic than single-agent ICIs [13]. Importantly, there are no reports of random controlled trials (RCT) comparing single-agent ICI with chemotherapy, and most of the available data are from small single-arm studies. In the clinical setting, the predictive utility of MMR status as a biomarker for the therapeutic effectiveness of ICIs is still contentious and unclear.

Therefore, we conducted this systematic review to establish consensus on the effectiveness and safety of PD-1/PD-L1 inhibitor monotherapy in EC patients. With regard to ORR, disease control rate (DCR), and adverse events (AEs) in dMMR and pMMR EC, respectively, our main objective was to completely quantify and compare the therapeutic advantages.

## Methods

The current study adhered to the guidelines established in the Preferred Reporting Items for Systematic Reviews and Meta-Analyses (PRISMA) [14]. Its protocol was formally registered in the International Prospective Register of Systematic Reviews (PROSPERO) database under CRD42023460363.

### Eligibility criteria

The Population, Intervention, Comparisons, Outcomes, and Study Design (PICOS) established the inclusion and exclusion criteria. Adult women with EC who were not infected with the HI virus were eligible for inclusion in studies. PD-1/PD-L1 inhibitors, such as EC immunotherapy using a single agent, were the subject of service interventions. The intervention may include pembrolizumab, nivolumab, dostarlimab-gxly, atezolizumab, durvalumab, and avelumab for this review. Any comparison group was acceptable, including standard therapy if possible or no interventions. DCR and AEs were the secondary outcomes, with ORR as the primary outcome. We excluded practice guidelines, letters, commentaries, books, editorials, review papers, case studies, pilot studies, and case reports.

### Search strategy

A thorough and methodical search was done on English electronic databases (EMBASE, PubMed, and Web of Science) between September 2016 and 2023. We searched for in-progress trials on the clinical trials register. In addition, we conducted a manual search of the bibliographies of the retrieved publications to identify any pertinent articles for inclusion in our review. The keywords we used as search terms included “endometrial cancer,” “endometrial tumor,” “nivolumab,” “pembrolizumab,” “atezolizumab,” “avelumab,” “durvalumab,” “dostarlimab,” “Nivolumab,” “PD-1 inhibitor,” “PD-L1 inhibitor,” and “immunotherapy”.

### Data screening and extraction process

Two authors searched through the Endnote X20-organized records' titles and abstracts to find relevant records. Following the pre-established criteria for inclusion, students were responsible for conducting an autonomous secondary screening of the entire text. Additionally, they were tasked with independently gathering data from the selected studies utilizing a structure specifically designed within a Microsoft Excel spreadsheet. Details about the author, the intervention, the publication year, the sample size, the setting, the study stage, and the results were retrieved. The third author was responsible for resolving any differences of opinion during the entire process. For each study, the requirements for inclusion in the meta-analysis were established.

### Quality of study

The present study utilizes the Methodological Index for Nonrandomized Studies (MINORS) as a framework for assessing the methodological quality of the research., which has eight items to gauge the study quality assessed quality [15]. Independently, two authors appraised the quality. Any differences of opinion were thoroughly discussed until an agreement was reached. Table 1 shows a summary quality score for the included studies.

**Table 1:** Characteristics and outcomes of included studies

Study/trial	Registration	Country	Phase	Sample size	Age	Treatment	Type of ICI	MMR population	Objective response rate		Disease control rate		Adverse event incidence		Minors
									overall	MMR	overall	MMR	overall	Grade 3 or higher	
AN Fader 2016	NA	USA	2	9	NA	Pembrolizumab	PD-1	dMMR	5/9	NA	NA	NA	NA	NA	13
GF Fleming 2017	NCT01375842	USA	1	15	61 (20–74)	Atezolizumab	PD-L1	pMMR (7) dMMR (1)	2/15	pMMR (1/7) dMMR (–)	4/15	pMMR (1/7) dMMR (–)	NA	NA	14
Dung T Le 2017	NCT01876511	USA	2	15	57 (24–92)	Pembrolizumab	PD-1	dMMR	8/15	NA	11/15	NA	NA	NA	14
Patrick A Ott 2017	NCT02054806	USA	1b	24	67 (34–87)	Pembrolizumab	PD-1	pMMR (18) dMMR (1)	3/23	pMMR (3/18) dMMR (–)	NA	NA	13/24	4/24	15
Kosei Hasegawa 2018	JapicCTI-163212	Japan	2	22	NA	Nivolumab	PD-1	dMMR (2)	5/22	NA	NA	NA	14/22	4.22	13
PA Konstantinopoulos 2019	NCT02912572	USA	2	31	NA	Avelumab	PD-L1	pMMR (16) dMMR (15)	5/31	pMMR (1/16) dMMR (4/15)	13/31	pMMR (5/16) dMMR (8/15)	22/31	6/31	14
YC Antill 2019	ACTRN12617000106336	Australia	2	71	67 (36–81)	Durvalumab	PD-L1	pMMR (36) dMMR (35)	18/71	pMMR (1/36) dMMR (17/35)	25/71	pMMR (7/36) dMMR (18/35)	NA	NA	15
Ana Oaknin 2020	NCT02715284	Spain	1b	71	64 (38–80)	Dostarlimab	PD-1	dMMR	30/71	NA	28/71	NA	46/71	9/71	15
Aurelien Marabelle 2020	NCT02628067	France	2	49	60 (20–87)	Pembrolizumab	PD-1	dMMR	28/49	NA	NA	NA	32/49	7/49	14
S Bellone 2021	NCT02899793	USA	2	24	NA	Pembrolizumab	PD-1	dMMR	14/28	NA	NA	NA	NA	12/24	14
DM O'Malley 2022	NCT02628067	USA	2	90	64 (42–86)	Pembrolizumab	PD-1	dMMR	38/79	NA	NA	NA	68/90	11/90	15
A Oaknin 2022	NCT02715284	Spain	1	264	65 (30–86)	Dostarlimab	PD-1	pMMR (142) dMMR (106)	69/264	pMMR (19/142) dMMR (46/106)	114/264	pMMR (19/142) dMMR (59/106)	196/290	48/920	15

*NA* not available, *PD-1* anti-programmed cell death protein 1, *PD-L1* anti-programmed cell death ligand 1, *MMR* mismatch repair

### Publication bias and heterogeneity

To reduce the possibility of bias, comprehensive searches have been conducted (both manually and electronically). According to guidelines for examining and evaluating the asymmetry of funnel plots in meta-analyses, we employed funnel plots to determine the publication bias [16], but we could not do so for other aspects of the analysis due to a lack of studies. The Higgins I2 test examined study heterogeneity with its accompanied p-value by the Cochrane Handbook criteria.

### Data synthesis

The included studies determined pooled ORR, DCR, and AEs for each patient. We further assessed the differences across tumor subgroups based on the MMR status. Where studies contain both pMMR and dMMR patients, an estimated association in ORR was OR with 95% CI. Texts and forest plots were used to present the results. P-values less than 0.05 indicate statistical significance in the sensitivity study, which was carried out using leave-one-out influence analyses. The data syntheses were produced using R-studio Version 1.1.383 (1999 Free Software Foundation, Boston, Massachusetts, USA: RStudio, PBC).

## Result

### Literature search

**Fig. 1:**
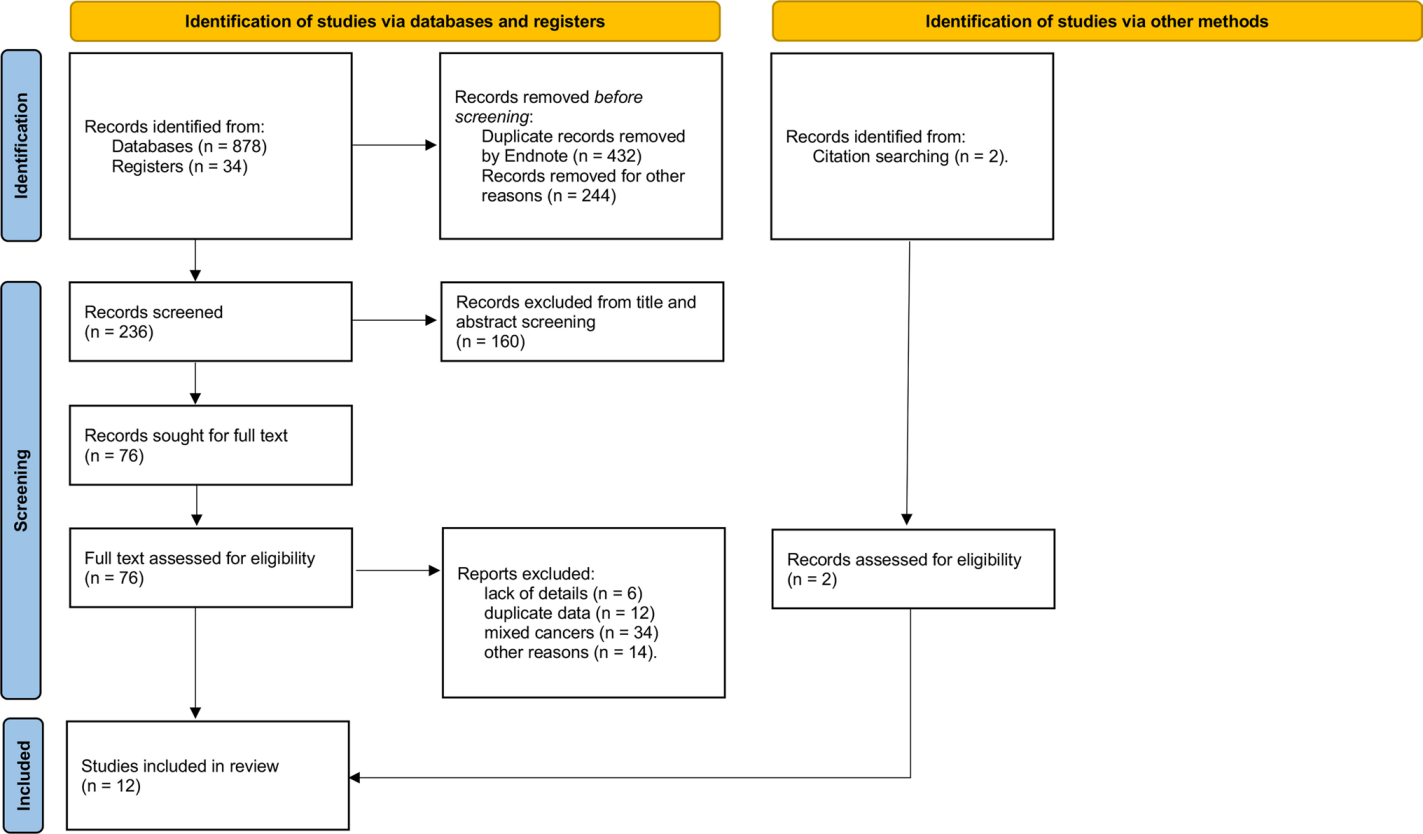
PRISMA flowchart of study inclusion and exclusion

By searching databases and the reference sections of relevant articles, 914 citations were found. 432 duplicates were removed electronically, with 244 studies excluded for study design, leaving 238 unduplicated and available citations. We evaluated the entire text and registration information of 78 papers for eligibility after eliminating 160 based on titles and abstracts that did not fit the criteria. Then, we eliminated 66 studies and included 12 studies. A PRISMA flowchart was reported in Fig. 1.

### Characteristics of included studies

Table 1 gives a summary of the features of the mentioned studies. Of the 12 presented studies [7, 8, 10, 17–25], three were conference abstracts, and the remaining nine were available full-text. The earliest study was published in 2016 [17], along with two full-text studies conducted by O’Malley and Oaknin in 2022 [22, 23]. Most of the studies (7/12) were conducted in the US. The most applied ICI was PD-1 inhibitor, specifically pembrolizumab, nivolumab and dostarlimab. With 685 individuals, all studies have 9–246 samples. Five studies included patients with both pMMR and dMMR status, but only three had enough samples to allow for between-group comparisons. Table 1 also includes the quality scores of the included studies.

### Quantitative synthesis

#### Objective response rate

**Fig. 2:**
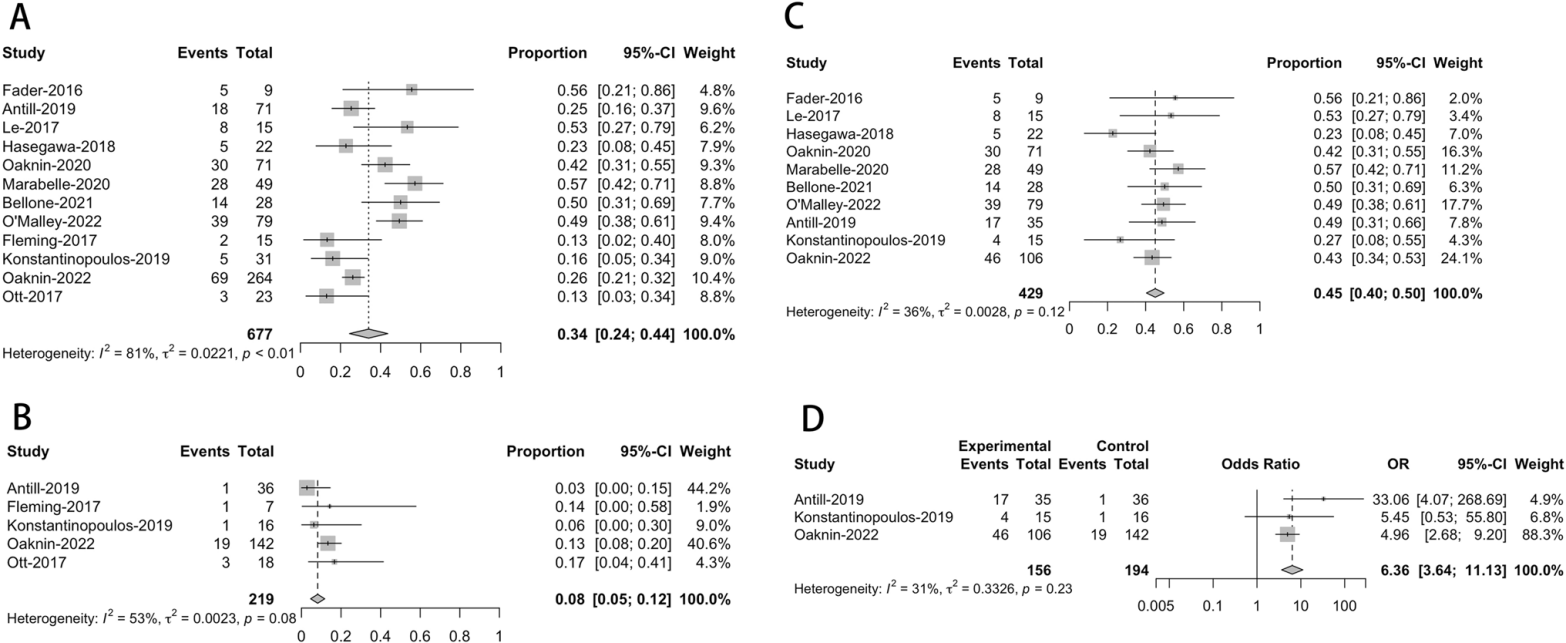
Forest plots of meta-analysis of effectiveness based on ORR. (**A** for overall ORR, **B** for pMMR patients, **C** for dMMR patients, **D** for meta-analysis of association)

The impact of single-agent immunotherapy with PD-1/PD-L1 inhibitors was examined in the twelve investigations, focusing on the ORR. As summarized in Table 1 and depicted in Fig. 2, the highest reported ORR was 57% (28 out of 49 patients) in the study by Marabelle in 2020 [8], while the lowest was 13% (2 out of 15 patients for Fleming in 2017 and 3 out of 23 patients for Ott in 2017) [10, 18]. Our meta-analysis observed significant heterogeneity in the combined ORR among patients with EC. The I2 statistic showed a value of 81%, and the Cochrane Q statistic gave a p < 0.05. Given this heterogeneity, we applied a quality effect model (QEM) to estimate % pooled ORR of 34% (95% CI = 24–44%, as shown in Fig. 2A).

We conducted a subgroup analysis based on the MMR status to better understand the source of heterogeneity and potential differences in efficacy. Our analysis, which was based on a fixed effect model (FEM) because there was no significant heterogeneity (p = 0.12, I2 = 36%, as shown in Fig. 2C), revealed a pooled ORR of 45% (95% CI = 40–50%) for patients with dMMR. In contrast, patients with pMMR status exhibited a pooled ORR of 8% (95% CI = 5–12%), as depicted in Fig. 2B, also based on a FEM. A meta-analysis was performed to investigate the relationship between PD-1/PD-L1 inhibitor immunotherapy's ORR and MMR status (dMMR and pMMR). This research used data from three studies and found that patients with dMMR status had considerably higher success rates with PD-1/PD-L1 inhibitors than those with pMMR status. The OR was 6.36, with a 95% CI ranging from 3.64 to 11.13, as presented in Fig. 2D.

#### Disease control rate

**Fig. 3:**
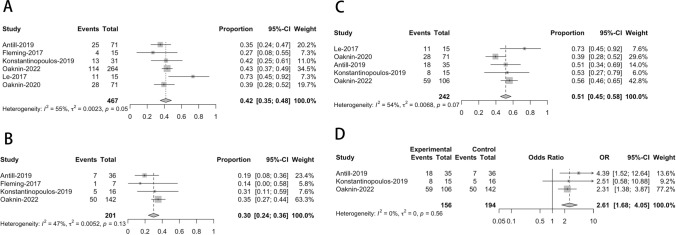
Forest plots of meta-analysis of effectiveness based on DCR. (**A** for overall DCR, **B** for pMMR patients, **C** for dMMR patients, **D** for meta-analysis of association)

Only six of the included studies reported the disease control rate (DCR). Most of these studies reported a DCR of less than 50%, except for Le’s study in 2017 [7], which had an impressive DCR of 73% (11 out of 15 patients). In our meta-analysis, we estimated an overall pooled proportion of DCR at 42% (95% CI = 35–48%) using a QEM, as shown in Fig. 3A.

In order to perform subgroup analysis taking into account patient availability with both pMMR and dMMR, the combined rate of Disease Control Rate (DCR) for people with pMMR was found to be 30% (with a 95% confidence interval ranging from 24 to 36%), while for people with dMMR, it was 51% (with a 95% confidence interval ranging from 45 to 58%). These estimates were derived using two FEM due to the absence of significant heterogeneity, as illustrated in Fig. 3B andC. Drawing upon insights gleaned from three distinct studies, we conducted a more comprehensive analysis to explore the connection between MMR status (specifically, dMMR and pMMR) and the Disease Control Rate (DCR) among patients receiving PD-1/PD-L1 inhibitor treatments. Figure 3D from our study shows no significant heterogeneity and that patients with dMMR had considerably higher efficacy of PD-1/PD-L1 inhibitors in terms of DCR than patients with pMMR (OR = 2.61, 95% CI = 1.68–4.05).

#### Adverse events

**Fig. 4:**
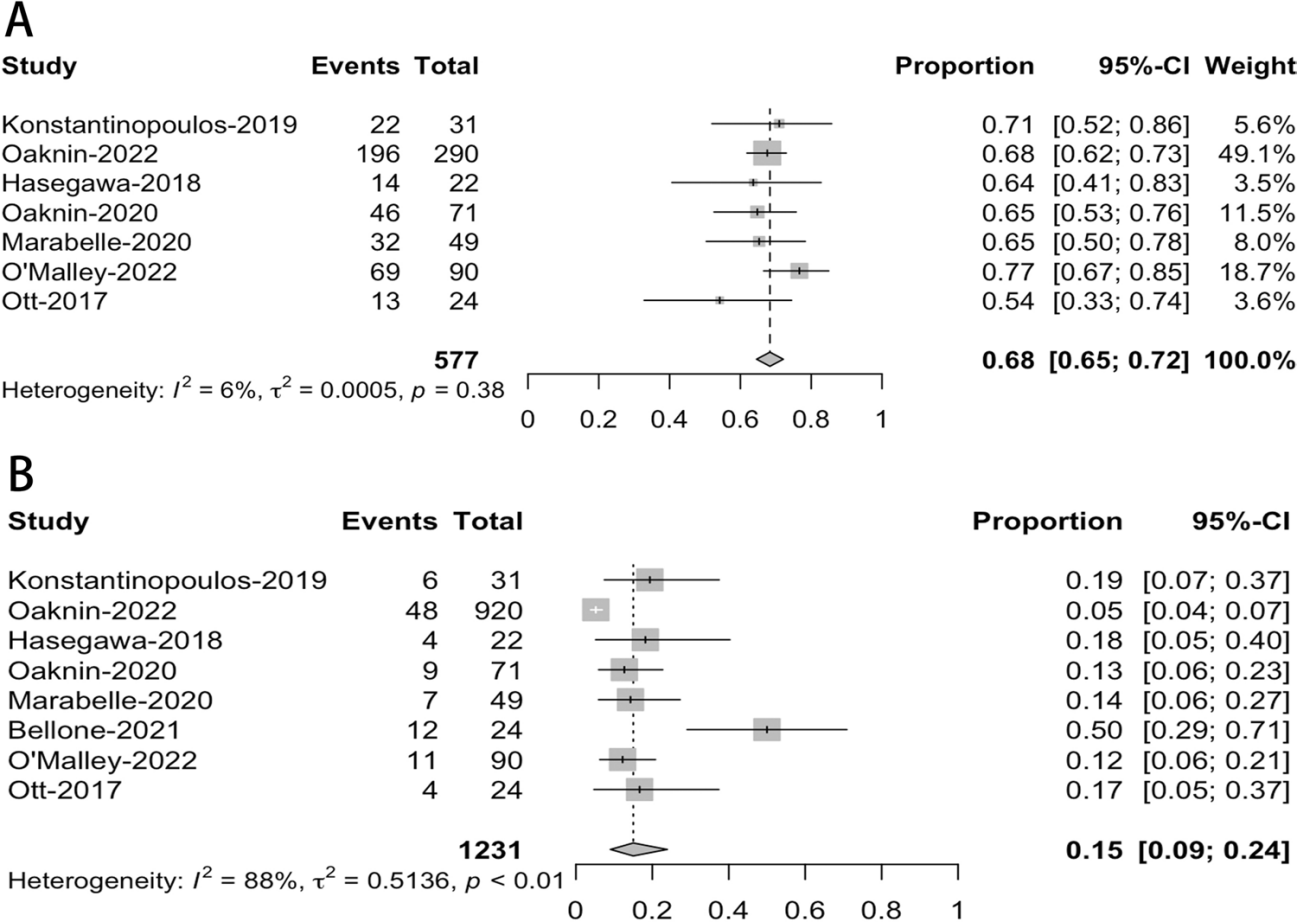
Forest plots of meta-analysis of AEs. (**A** for overall AEs, **B** for grade 3 or higher)

The analysis examined adverse events (AEs) across seven studies that involved patients with esophageal cancer (EC) participating in immunotherapy trials featuring PD-1/PD-L1 inhibitors. The overall proportion of EC patients receiving immunotherapy who developed AEs was 68% (95% CI = 65–72%). The Cochrane Q-statistic had a *p*-value greater than 0.05, and the I^2^ had a value of 6%, indicating that heterogeneity was not significant, as shown in Fig. 4A.

All eight trials examined adverse events (AEs) of grade 3 or higher in PD-1/PD-L1 inhibitor-treated EC trials. 15% (95% CI = 9–24%) of all adverse events (AEs) in EC patients receiving immunotherapy had a grade 3 or above. The Cochrane Q statistic's p-value was less than 0.05, and Fig. 4B's I^2^ value was 88%, demonstrating that heterogeneity was determined to be significant.

#### Publication sensitivity analysis

**Fig. 5:**
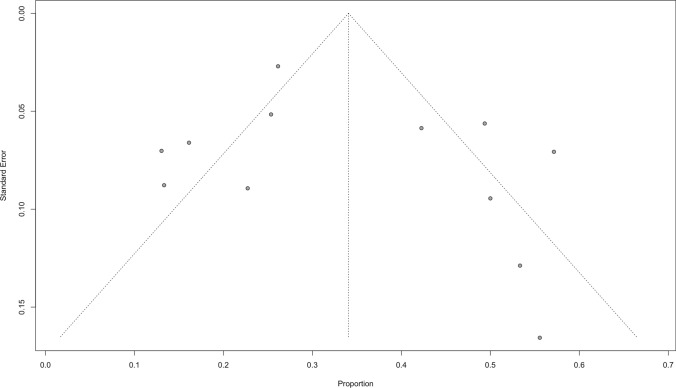
Funnel plot for publication bias of overall ORR

**Fig. 6:**
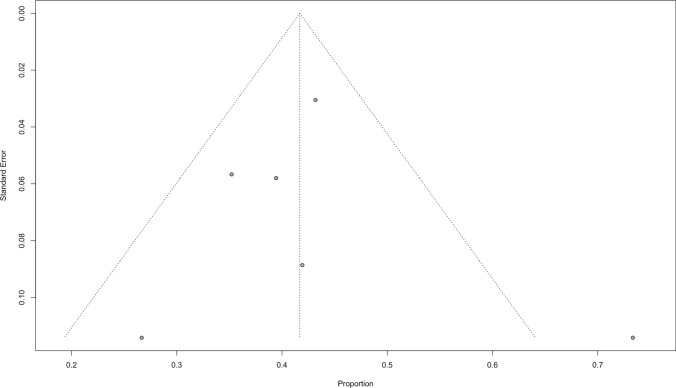
Funnel plot for publication bias of overall DCR

**Fig. 7:**
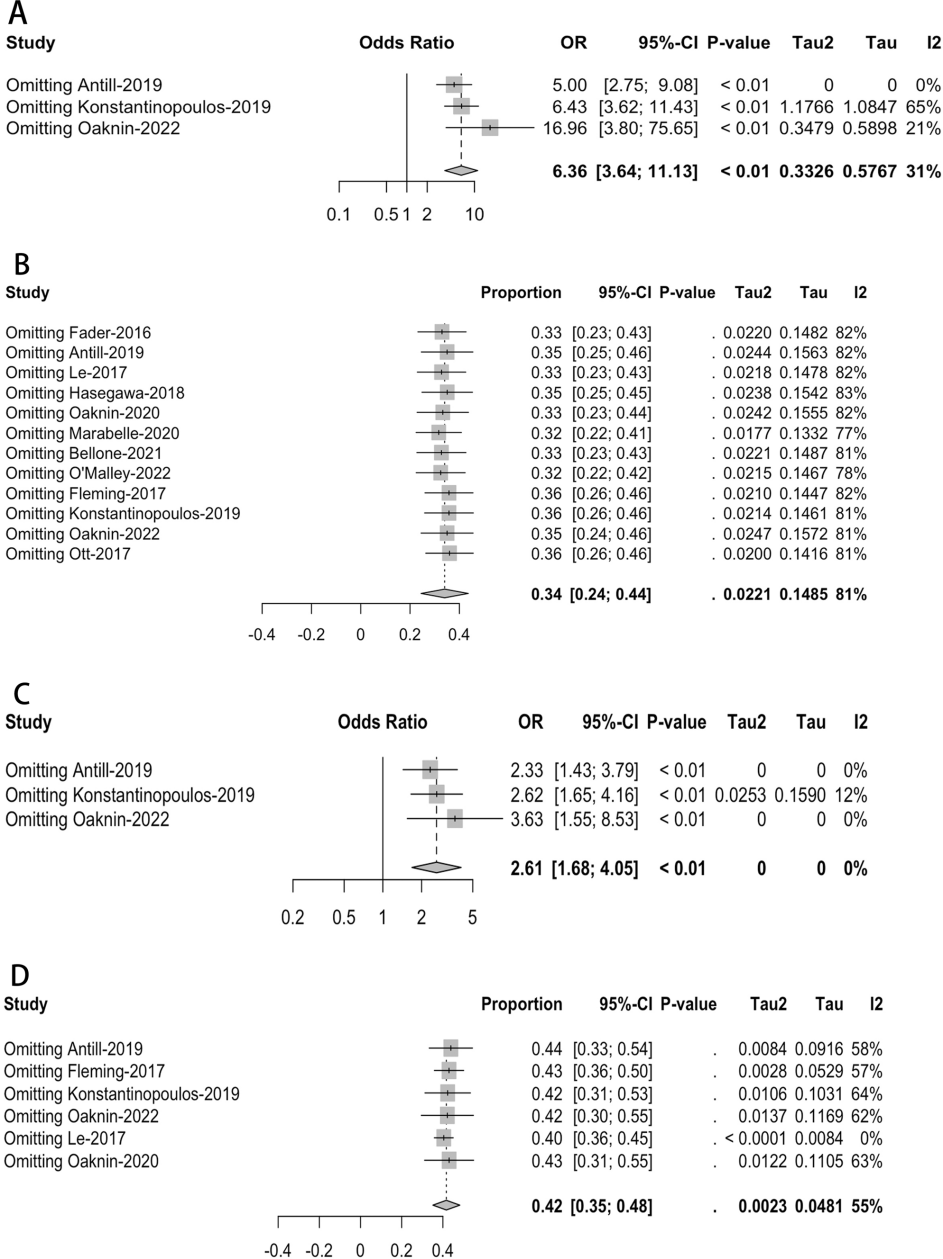
Forest plots for influence analysis. (**A** for meta-association of ORR, **B** for estimation of overall ORR, **C** for meta-association of DCR, **D** for estimation of overall DCR)

Funnel plots were employed to evaluate publication bias, but they failed to find any conclusive evidence of it in Fig. 5 and Fig. 6 for overall ORR and DCR. We conducted an impact analysis to evaluate the longevity and resilience of our combined safety (AEs) and efficacy (ORR, DCR) findings. The analysis demonstrated that leave-one-out influence analyses did not significantly alter variations in the pooled results for ORR sensitivity. Similar stability was observed in the other analyses presented in Fig. 7, with the range of variation falling within the effect size range, and the results remained statistically significant. The findings show that most of our aggregated results for various outcomes were largely steady, with only a minor influence from a single study.

## Discussion

Since its processes and effects were discovered in the last ten years, tumor immunotherapy has become a novel therapeutic technique in cancer research [26]. Much of the research in the field of ICIs has been centered around PD-1 and PD-L1 inhibitors. As a result, many research projects have been carried out to improve and validate these ICIs in the context of different cancers, including ECAs. a result, this systematic review provided a thorough summary of the PD-1/PD-L1 inhibitor immunotherapies used in earlier clinical trials and assessed the efficacy and safety information related to utilizing PD-1/PD-L1 inhibitors as stand-alone immunotherapy in EC patients by a meta-analysis.

Based on the meta-analysis's findings, It was known that PD-1/PD-L1 inhibitors have an ORR of 34%. (95% CI = 24–44%), DCR was 42% (95% CI = 35–48%). When evaluating the efficacy of clinical trials, an ORR greater than 30.0% is considered an appropriate endpoint for single-arm trials showing the revolutionary activity of single-agent cancer therapy [27]. So, according to our meta-analysis, the efficacy was statistically significant in women with advanced EC. ORR is frequently utilized as a substitute clinical endpoint for evaluating innovative targeted medicines for fast approval because it does not demonstrate long-term survival outcomes, which are the gold standard for assessing direct therapeutic efficacy. A specified response to treatment, such as the total removal of tumors (complete response) or the absence of any residual tumor following treatment, is what the ORR measures in patients. [27–29]. It helps with clinical practice decision-making and can be used to obtain regulatory approval. It is a significant indicator of the effectiveness of the treatment. Furthermore, DCR considers individuals with stable disease in addition to those who have shown a complete or partial response [30]. This is especially true when treating conditions when the main objective of therapy is to stabilize the illness rather than completely eradicate it, as is the case, particularly when treating recurrent EC. Thus, we have significant evidence for the clinical effectiveness of single-agent PD-1/ PD-L1 inhibitors in treating advanced EC.

Subgroup analysis showed that among dMMR patients with higher efficacy, MMR status could predict the effectiveness of single-agent PD-1/PD-L1 inhibitor immunotherapy. The pooled ORR for EC patients with dMMR was found to be 45% (95% CI = 40–50%), whereas it was 8% (95% CI = 5–12%) for EC patients with pMMR. The pooled proportion of DCR based on the availability of patients with pMMR was 30% (95% CI = 24–36%), and that of patients with dMMR was 51% (95% CI = 45–58%). Both the meta-analysis associations of ORR and DCR were significantly positive when dMMR patients compared with pMMR patients (OR = 6.36, 2.61, 95% CI = 3.64–11.13, 1.68–4.05, respectively). We acknowledge that prospective RCTs comparing single-agent ICI with non-ICI therapies and adequately robust RCTs establishing differences in survival outcomes depending on MMR status are required to establish the predictive utility of MMR status. According to the data, PD-1/PD-L1 inhibitor monotherapy exhibited substantial efficacy in the dMMR group, which should not be forgotten. A hypermutation in genomic microsatellites known as microsatellite instability is brought on by deficiencies in mismatch repair. Therefore, Future trials of advanced EC should incorporate MMR status as a stratification factor, which should be evaluated prospectively. More translational study is urgently required to determine the cause of dMMR and enable the classification of EC into several dMMR molecular subtypes.

68% of patients (95% CI = 65–72%) reported having all adverse events related to AEs. In 15% of patients (95% CI = 9–24%), grade three or higher adverse events were reported. The most common adverse reaction at any level was fatigue, followed by nausea, anaemia, diarrhoea, and hypothyroidism. Even though adverse reactions were observed in more than 60% of cases, Grade 1–2 adverse reactions typically did not cause patients to have serious problems. According to the study, patients' motivation to continue receiving therapy is generally not affected by the discomfort brought on by these side effects [31]. However, it is still important to improve clinical patient acceptability and comfort.

Furthermore, beyond exploring single-agent immunotherapies, clinical trials have actively investigated combination therapies for EC. Among these, one prominent combination therapy involves using pembrolizumab with lenvatinib, which recently received accelerated approval from the US FDA for EC cases that haven't progressed after previous treatments and don't exhibit dMMR [32]. Lenvatinib works against several targets, including the KIT proto-oncogene, receptor tyrosine kinase, fibroblast growth factor receptors, transfection-related rearrangement, and platelet-derived growth factor receptor alpha, which may trigger an immunological response [33]. The rationale is to boost anticancer immune response while also demonstrating anti-angiogenic properties. This approach has the potential for a synergistic effect, slowing down tumour growth and potentially improving patient prognosis. However, it's important to note that combining immunotherapy with targeted agents may lead to heightened toxicity and potentially serious adverse effects. In order to make sure that any adverse effects are properly treated, the safety and effectiveness of this combination drug are being closely monitored and examined in ongoing clinical trials.

Finally, we must point out a few limitations of our systematic review. First, this review’s primary research was restricted to a few internet databases with a small geographic scope, some of which may have been underrepresented. Second, only English-language papers were considered for this review, which might have left out studies available in other languages. In addition, the review did not include any grey literature because it might have affected the intervention's estimated magnitude.

## Conclusion

This comprehensive review and meta-analysis revealed the overall ORR and DCR after monotherapy immunotherapy with PD-1/PD-L1 inhibitors in patients with EC. Compared to pMMR patients, EC patients with dMMR showed considerably improved ORR and DCR. ICIs were considered safe because grade 3 or worse AEs only happened in 15% of instances. Clinically, these insights can improve therapy approaches by considering detailed patient characteristics. Finalizing patient profiles and identifying predictive biomarkers is crucial to promoting personalized medicine, specifically advanced EC and dMMR patients.

## Data Availability

The original data about the research have been included in this study.
